# Slack in the infrastructure of intensive care units: resilience management in the post-pandemic era

**DOI:** 10.1186/s12913-023-09495-4

**Published:** 2023-06-06

**Authors:** Carlos Emilio Stigler Marczyk, Tarcisio Abreu Saurin, Iamara Rossi Bulhões, Riccardo Patriarca, Federico Bilotta

**Affiliations:** 1 Construction and Infrastructure Post-Graduate Program, Universidade Federal do Rio Grande do Sul, Av. Osvaldo Aranha, 99, Porto Alegre, CEP 90035-190 Brazil; 2 Industrial Engineering Post-Graduate Program, Universidade Federal do Rio Grande do Sul, Av. Osvaldo Aranha, 99, Porto Alegre, CEP 90035-190 Brazil; 3 Department of Mechanical and Aerospace Engineering, Sapienza University of Rome, Via Eudossiana 18, Rome, 00184 Italy; 4 Department of Anesthesiology, Critical Care and Pain Medicine, Sapienza University of Rome, Via Eudossiana 18, Rome, 00184 Italy

**Keywords:** Slack, Intensive care units, Resilience engineering, Infrastructure, COVID-19

## Abstract

**Background:**

Although slack is an asset to resilient hospitals, it is usually explicitly discussed only in terms of the quantity and quality of beds and staff. This paper expands this view by addressing slack in four infrastructures of intensive care units (ICUs) (physical space, electricity supply, oxygen supply, and air treatment) during the COVID pandemic.

**Methods:**

The study occurred in a leading private hospital in Brazil, aiming at the identification of slack in four units originally designed as ICUs and two units adapted as ICUs. Data collection was based on 12 interviews with healthcare professionals, documents, and comparison between infrastructures and regulatory requirements.

**Results:**

Twenty-seven instantiations of slack were identified, with several indications that the adapted ICUs did not provide infrastructure conditions as good as the designed ones. Findings gave rise to five propositions addressing: relationships intra and inter infrastructures; the need for adapted ICUs that match as closely as possible the designed ICUs; the consideration of both clinical and engineering perspectives in design; and the need for the revision of some requirements of the Brazilian regulations.

**Conclusions:**

Results are relevant to both the designers of the infrastructures and to the designers of clinical activities as these must take place in fit-for-purpose workspaces. Top management might also benefit as they are the ultimate responsible for decision-making on whether or not to invest in slack. The pandemic dramatically demonstrated the value of investing in slack resources, creating momentum for this discussion in health services.

**Supplementary Information:**

The online version contains supplementary material available at 10.1186/s12913-023-09495-4.

## Introduction

Intensive care units (ICUs) were at the front-line of the COVID-19 pandemic^1^, having their human and material resources stretched. The high incidence of patients with the same disease implied in a peak of demand for certain resources (e.g., oxygen, isolation rooms, drugs), raising questions on the slack resources to cope with extreme events. According to Bourgeois [1] “slack is a cushion of actual or potential resources which allows an organization to adapt successfully to internal pressures for adjustment or to external pressures for change in policy”. Slack resources can take many forms such as financial reserves, extra space, surplus of materials, workers on standby, redundant equipment, and generous time margins, among others [2]. This article explores the contribution of slack to resilient healthcare, defined as the “ability of the healthcare system to adjust its functioning prior to, during, or following changes and disturbances, so that it can sustain required performance under both expected and unexpected conditions” [3]. Similarly, Wiig et al. [4] define resilient healthcare as “the capacity to adapt to challenges and changes at different system levels, to maintain high quality health care”. Four organizational potentials are commonly associated with resilient health services, namely the potentials of monitoring, anticipating, responding, and learning [5]. Disconzi and Saurin [6] argue that designing slack resources and practices is a key principle for the design of resilient systems, being connected mostly to the responding resilience potential. Anderson et al. [7] add that mismatches between capacity and demand, which usually indicate insufficient slack, are common triggers of resilient performance in health services.

In hospitals, slack is usually explicitly approached only in terms of the number and quality of beds and workers [8], which have been major issues in ICUs during the pandemic. Less attention has been devoted to the infrastructure of ICUs, which, if insufficient, might amplify the consequences of lack of beds and staff. This article explores slack in four infrastructures of ICUs during the pandemic, namely physical space, electricity supply, oxygen supply, and air treatment. Physical space (e.g., size, form, layout, visibility) sets constraints for the use of the other infrastructures and for all care and administrative activities [9]. Electricity and oxygen supply must be highly reliable given their patient safety implications [10, 11]. Air treatment is concerned with air quality, including the creation of air pressure gradients to prevent the spread of contaminated air [12]. While architectural design can contribute to air quality [13], this paper is limited to the engineered heating, ventilating, and air conditioning (HVAC) infrastructure.

Although there are other relevant infrastructures such as those related to water supply and information technology, their consideration would imply a scope too large for the present study. Moreover, the selected infrastructures encompass key issues for the ICUs during the pandemic, being associated with events of public knowledge such as overcrowded facilities and the lack of oxygen supply, both in high and medium-income countries [14, 15].

The need for some slack resources such as the use of power generators in hospitals, is foreseen in regulations that establish the minimum requirements that should be met by healthcare facilities [16]. However, no regulation can anticipate all scenarios that demand slack and therefore the continuous improvement of regulations inevitably occurs, in part, based on learning from unexpected shocks [17]. In many countries, the provision of care during the pandemic was uncharted territory that posed the hardest test ever for slack in health services, challenging the assumptions of designers and regulators.

Against this background, the research question addressed by this study is stated as follows: how can the design of slack in the infrastructure of ICUs support their resilient performance in the post-pandemic era? This question was investigated based on a case study of the ICUs of a leading private hospital in Brazil, one of the countries most affected in terms of number of deaths; almost 700,000 up to March 2023 [18]. The case study allowed to confront the reality of the selected infrastructures against the needs perceived by staff and established by regulations, besides shedding light on their interdependences. This last point is worth highlighting as no previous study has jointly investigated the four infrastructures, despite their relationships. Findings set a basis for propositions aimed at the design of slack in the post-pandemic era.

## Background

### Slack: benefits, drawbacks, and practices

Slack is associated with benefits to resilience [2], safety [19], innovation [20], and sustainability [21]. It can also affect service quality dimensions such as responsiveness, namely the provision of prompt services to clients [22]. The rationale for these benefits is that slack dampens the propagation of variability, allowing performance adjustment that characterizes resilient performance, protecting people from safety hazards, allowing cycles of trial and error that are typical of the innovation process, and providing extra human and financial resources to invest in sustainable solutions. In the ICU context, Bueno et al. [23] conducted a systematic review and concluded that the use of slack was ubiquitous in the design of quality and safety improvement interventions.

Slack also has downsides, especially when it involves the introduction of extra elements (e.g., people, equipment, procedures) into a system. The addition of elements increases the number and nature of interactions in a complex system, potentially giving rise to unintended consequences [24]. Further, slack can encourage complacency of workers who over-trust the extra safety barriers [25] as well as it can mask wastes. Saurin and Ferreira [26] exemplify waste implications in a hospital ward where the unlevelled demand for medications during the day required the use of two automated dispensing cabinets; once demand was levelled, one of the cabinets proved to be unnecessary. Thus, deciding the proper amount of slack is not trivial as it means finding a balance between the benefits and drawbacks of slack. This decision depends on the costs of slack resources and the local consensus of what counts as acceptable risk [27].

While slack might be deployed through several practices, the most common is the use of redundancies, defined as resources in addition to the minimum necessary to perform a function [28], or more than one resource performing a required function [29]. Another common slack practice is the use of margins of manoeuvre. Stephens et al. [30] describe two common types of margins: (*i*) defensive, characterized by restricting other units’ actions or borrowing other units’ margin e.g., the suspension of elective surgeries during the pandemic created margin for ICUs; and (*ii*) autonomous, characterized by local reorganization or adaptation of resources in time of need – e.g., process improvement initiatives that release labour or space.

Finally, not all slack resources are deliberately devised to serve as slack. Under certain circumstances, any resource can play an opportunistically role as slack - e.g., an inflatable children toy in a swimming pool can work as a buoy to prevent drowning. [2] use the designed versus opportunistic distinction along with other categories (e.g., whether slack is a legal requirement, the visibility, and durability of slack) in their taxonomy of slack resources.

### Slack in the ICU infrastructure

In this section, key aspects of slack in the selected ICU infrastructures are presented, including an overview of the Brazilian legislation. Regarding physical spaces, ICUs are often stressful, noisy, and crowded workplaces [31]. A frequent topic of discussion in prior studies refers to the ICU form and layout, especially concerning the location of the nursing station, either centralized, decentralized, or hybrid [9].

In Brazil, regulation RDC 50/2002 sets the requirements for the planning, elaboration, assessment, and approval of healthcare building design [16]. This regulation includes requirements for ICUs such as those related to the size of spaces. For example, RDC 50/2002 specifies that beds should be placed at least 1.0 m away from the lateral walls, and at least 1.2 m from the back wall – these dimensions correspond to an overall patient room of approximately 10 m^2^. Size requirements of this regulation differ from those of similar regulations in developed countries. For example, the size of ICU patient rooms in the U.S. should be at least 18 m^2^ [32].

Regulation RDC 50/2002 also covers the ICU electricity supply. It requires an independent, emergency electricity network, for three ICU spaces: nursing station, medical prescription room, and patient room. The same regulation defines the maximum tolerable recovery time in case of power outages – e.g., equipment for mechanical ventilation, widely used in ICUs, must be automatically reactivated by an emergency source of power in no more than 15 s when there is a drop of 10% or more of the nominal voltage, being available for at least 24 h [16].

As for the infrastructure of oxygen supply, studies of countries like China [33] and Poland [10] describe how it was severely stressed during the pandemic. In Brazil, the surveillance branch of the ministry of health demanded weekly reports from manufacturers and distributors of medical oxygen to monitor their overall inventory levels [34]. RDC 50/2002 sets requirements for oxygen supply for each ICU bed such as the availability of at least two points of use and minimum flow rate of 60 L/minute. Moreover, Brazilian standard NBR 12,188/2016 [35] posits that, in ICUs, designers should consider that at least 80% of the points of oxygen use will be working concurrently. These three criteria (number of points of use, flow rate, and concurrent use) set the basis for designing the oxygen distribution network and the storage capacity, usually composed of tanks and/or cylinders. ICUs must count on an emergency reserve storage, independent on the central reserve storage for the hospital as a whole [35].

Regarding the air treatment infrastructure, it is crucial for the prevention of COVID infection as the virus is transmitted through aerosols and can travel fairly large distances through the air [36]. RDC 50/2002 establishes that HVAC utilities of ICUs must be connected to an electrical circuit of emergency, highlighting the relationships between infrastructures [16]. Another exemplar interdependence occurs between HVAC and oxygen supply. In the pandemic there have been greater concerns with fires in ICUs due to oxygen saturation stemming from small leakages that are to some extent unavoidable [37]. Frequent air renovation allowed by proper HVAC can reduce this risk. For more detailed requirements, RDC 50/2002 refers to Brazilian standards NBR 16,401 (air conditioning) and NBR 7256 (air treatment in healthcare facilities). This last standard was revised during the pandemic, re-defining requirements related to air temperature, humidity, purity, renovation, flow, pressure, and types of air filters, besides demanding redundancies in the sources of cooling and heating [34].

## Research method

### Research strategy

Case study was the adopted research strategy [38] as it offers an opportunity for understanding a complex, recent and underexplored topic (i.e., slack in the infrastructure of ICUs during the pandemic) in a real context. The studied ICUs were part of a private hospital in Brazil, which in 2020 had 481 in-patient beds, approximately 4,000 employees and 4,000 accredited physicians who could choose this hospital for treating their patients. This hospital was an important hub of COVID patients, noting that private hospitals account for approximately 52% of the ICU beds in Brazil [39]. This hospital is located in a 1.5 million people capital city and is widely known as a leading healthcare organization in the country in terms of quality of care. The hospital holds certifications by the Joint Commission International as well as ISO 9001/2015^2^ [40] for the administrative and care processes. As such, this hospital was clearly above the national average in terms of financial and technological resources, which could be an asset for the provision of slack resources. Therefore, if the scarcity of slack resources was a reality for this hospital during the pandemic, the scenario for most other private and public hospitals would probably be even worse.

Internal validity was achieved by following good practices of case-based research [38, 41], namely: the use of multiple sources of data, thus allowing for triangulation; the development of a database that could be revisited anytime for re-interpretation of data; and the definition of a unit of analysis, which was slack in the ICU infrastructure. The study proposal, including all data collection and analysis protocols, was approved by the research ethics committee of the hospital.

### The studied ICUs

COVID patients were hospitalized both in four out of the five adult existing ICUs (hereafter referred to as designed ICUs) and, during the most critical moments, in two areas turned into improvised ICUs, namely the ward for bone marrow transplanted patients and the recovery room of the surgical unit (hereafter referred to as adapted ICUs). The bone marrow ward was used only during the first peak of the pandemic in April 2020. The bone marrow transplanted patients were transferred to a surgical ward that was free as elective surgeries had been suspended during the pandemic. The surgical recovery room was used only in the highest peak in March/April 2021. Table 1 presents the main characteristics of the studied units, built over several years and subject to the regulations and best practices of the time. The main hospital building dates from 1921 and has been expanded and renewed multiple times.

**Table 1:** Characteristics of the hospital units used for the hospitalization of COVID patients

**Designed ICUs**	**Inauguration year**	**Number of beds**	**Staff**
ICU 1	1990	11	1 doctor (DR), 2 nurses (N), 7 nurse technicians (NT), 1 physical therapist (PT)
ICU 2	2000	10	1 DR, 1 N, 7 NT, 1 PT
ICU 3	2006	10	1 DR, 1 N, 7 NT, 1 PT
ICU 4	2017	17	2 DR, 2 N, 10 NT, 2 PT
Total for the designed ICUs	-	48	5 DR, 6 N, 31 NT, 5 PT
**Adapted ICUs**	**Inauguration year**	**Number of beds**	**Staff**
Surgical recovery room	2001	30	3 DR, 3 N, 21 NT, 3 PT
Bone marrow ward	2017	22	2 DR, 2 N, 12 NT, 2 PT
Total for the adapted ICUs	-	52	5 DR, 5 N, 33 NT, 5 PT
**Total ICU beds**	-	100	-

**Note**: minimum number of staff members per shift. There are three daily shifts

### Data collection

Semi-structured interviews, documents, and regulations were the main sources of data. Four criteria were used for selecting the interviewees: at least one year of experience at the hospital; experience at the ICUs during the pandemic; awareness of the purpose and overall functioning of the selected infrastructures; and availability to participate in the study. We were able to recruit 12 interviewees who met these criteria. Their average experience at the hospital ranged from 3.5 to 24 years (mean 11.7 years). The panel of interviewees encompassed one doctor (DR, was responsible for the planning of the ICU medical roster, and is currently the hospital medical director), three nurses (N1, N2, N3), three nurse technicians (NT1, NT2, NT3), two physical therapists (PT1 and PT2), and two engineers who had management positions at the infrastructure management department (E1, E2). Considering the population for each professional category of caregivers (see Table 1), the panel was more representative of nurses (27% of the nurses were interviewed) and less representative of nurse technicians (5% of the total). This is not a drawback since nurses are well-known for their leadership role in health services^3^ [42], which is relevant for the co-ordination of activities across hospital units such as between ICUs and the infrastructure department. N3 was interviewed twice: firstly, for data collection on the slack resources and, at the end of the study, for obtaining her feedback on the accuracy of the findings and practical implications. She was chosen for this final interview due to her position as head of the hospital quality and safety management system. N1 and N2 also had leadership positions, respectively as ICU chief-nurse and hospital chief-nurse.

The interviews were conducted by CM who had 25 years of experience as the infrastructure manager of the hospital, under the guidance of TAS and IRB who are experienced researchers. At the time of data collection, CM had already left his position at the hospital more than one year earlier. This researcher is recognized as an expert in hospital infrastructure, being a member of national regulatory committees, offering consulting services, and lecturing on this topic in academic and professional events. This implied in first-hand and technical knowledge of the investigated topics. The interviewer used a question guide (supplementary file 1), aimed at understanding the manifestations of slack in the selected infrastructures.

All interviews occurred during working hours, were audio recorded, lasted from 20 to 60 min (9 h of recordings, mean 45 min), were transcribed verbatim (50,800 words), and anonymised prior to the analysis. Interviews were discontinued when reports started being clearly convergent and data seemed to suffice for answering the research question, indicating that the data saturation criterion had been met. The number of interviews (12) is equal to that suggested by several studies as the threshold of data saturation [43, 44].

Documentary analysis involved different types of designs from the studied units (e.g., architecture, electricity), and records of electricity and oxygen consumption during the pandemic. Regulation RDC 50/2002 was also consulted, allowing for a comparison between the minimum regulatory requirements and the existing resources.

### Data analysis

A thematic analysis was carried out for making sense of the transcripts from interviews and documents, following the recommendations by [45]. The familiarization step involved reading the raw data several times in order to gain understanding of the recurring themes. Next, the five themes defined upfront (i.e., the four slack infrastructures and problems that demanded slack) were imposed on the data by the researchers. In the coding step, excerpts of text were tagged according to the themes. We looked for excerpts of text that matched the slack definition by [1] presented in the Introduction. Thus, we coded these excerpts as slack instantiations, corresponding to manifestations of slack in a particular infrastructure, at a certain moment and place. When appropriate, the description of slack identified from the interviews was complemented by a quantitative measure, defining slack as the share of resources that exceeded the regulatory requirement. For example, a patient room with 19.0 m^2^ was deemed to have 90% slack in relation to a regulatory requirement of 10.0 m^2^. The first author conducted a preliminary coding, followed by a careful review by the second and third authors – all these authors read the full transcripts of interviews. In the charting step, coded data were presented (see Results) in four tables, one for each slack infrastructure. The mapping and interpretation step involved the joint analysis and discussion of the findings using the lens of resilience engineering.

Based on these steps, propositions were developed in accordance to the inductive case study approach. The development of propositions starts from the identification of patterns in data and the creative development of explanations for those patterns [46]. The propositions are intended to be relevant to further theory development, theory testing, and for guiding action in the design of the selected infrastructures, with the objective of supporting resilient performance to cope with future pandemics.

## Results

### Slack in the physical space

Table 2 presents the identified instantiations of slack in the ICUs´ physical spaces. These examples are related not only to the size of the spaces but also to their layout and purpose. Instantiation SP1 is concerned with the visibility of patient rooms from the nursing station, which lowers the frequency of access to the rooms. The following remark from the interviewed doctor (DR) illustrates this point: “*the least we stay in the patient room the better…the usual procedure of checking patient conditions at the bedside was not safe”*. Nurse N3 added that “*we put the computers at the corridors to avoid entering the patient room”*, while the physical therapist PT2 stated that the circular layout of the ICUs “*allowed to see all patients at the same time”*. However, instantiation SP2 conveys that visibility might also be obtained if patient rooms are arranged linearly, despite “*the need for visualizing patients one at a time from the small nursing stations created near the entrance door to the rooms”*, as reported by nurse N4.

**Table 2:** Slack in the physical spaces

Code	Slack instantiation	N	Problems that created the need for slack	Designed ICU / Adapted ICU
SP1	Centralized nurse stations at ICUs 1, 2, and 3, surrounded by patients´ room in a circular layout. This facilitates the visualization of patients from the nurse station.	9	Visibility from the nurse station reduced the need for staff entering patient rooms, reducing risk of infection	Designed
SP2	Despite the linear shape of ICU 4, there are small decentralized nursing stations at the corridors, near the entrance door of the rooms, allowing visibility to patients	9	Same as above	Designed
SP3	Nursing stations in all ICUs are larger (from 67–300%) than the regulatory requirement	5	The seriousness of COVID patients required more resources such as staff, equipment and supplies, demanding larger spaces	Designed
SP4	Patient rooms in all ICUs are larger (from 30–93%) than the regulatory requirement	10	Same as above	Designed
SP5	ICU 4 rooms were the largest and had pendants that facilitated the arrangement of people and surgical equipment	2	Emergency surgeries (e.g., deliveries) sometimes needed to be carried out in the ICU	Designed
SP6	Records of the plan of care and patient condition were made on the walls of the ICU rooms, for the subsequent transcription to electronic records	1	Making records at the bedside, either electronically or on paper, implied greater exposition to contamination	Designed
SP7	Blind mobile wall separating beds at the bone marrow ward, aiming at patient privacy	6	Lack of privacy for patients at the bone marrow unit	Adapted
SP8	Installation of additional doors and mobile dividing walls at the recovery room in order to separate clean (i.e., areas for donning PPE) from contaminated areas	6	Open space at the recovery room increased opportunities for contamination	Adapted
SP9	Pharmacies were installed within the recovery room and the bone marrow ward	7	ICU pharmacy was distant from the adapted ICUs	Adapted

**N**: number of interviewees who reported the slack instantiation

**SP**: slack in physical space

The extent of the necessary slack in the physical spaces underlined SP3 and SP4. Although the size of all nursing stations and patient rooms of the designed ICUs exceeded the regulatory requirements, the interviewees pointed out that the slack at the higher end of the observed ranges (i.e., 300% for nursing station and 93% for patient room) was the desired condition to make a difference. Nurse N3 reported that “*several pieces of equipment can be necessary for these patients such as dialysis machine, invasive and non-invasive ventilatory equipment, extracorporeal membrane oxygenation, and several infusion pumps; further, we need to move around the patient to operate these technologies”.* Nurse N4 stressed the benefits of ICU 4, whose patient rooms were 93% larger than the regulatory requirement (in comparison to 30% at ICU 1): “*we can place everything in the room at the same time….in the other ICUs, the rooms do not accommodate the dialysis machine…besides, new treatments usually imply in additional life-supporting technologies at the bedside”.* The physical therapists appeared to be particularly satisfied with the larger size of ICU 4, as they bring their own devices into the patient room for providing care. PT2 remarked that in the ICUs other than ICU 4, she could not put an armchair (patients need to be on the chair for rehabilitation) in the room without removing the bed to the outside.

SP5 demonstrates the life-saving implications of slack in the patient rooms, during a caesarean section. According to nurse N3, “*we had a pregnant patient with COVID…her condition quickly deteriorated and intubation was the only possible course of action; the medical team decided to make the caesarean section in the ICU because the patient had no conditions to be transported to the surgical unit…luckily this situation occurred at the more spacious ICU 4; I have no doubt this caesarean would have been much more difficult, if not impossible, in the other, smaller ICUs*.“ In turn, instantiation 6 shows that opportunistic slack can also play out in the designed ICUs, as stated by nurse N3: “*people were using the window glass to make patient records…this avoided the handling and exchange of contaminated pieces of paper and also implied in rethinking our work organization.“*

As for the adapted ICUs, the additional bed capacity they provided came at a cost in terms of safety and efficiency. The interviewed doctor stressed the drawbacks of improvising ICU care at the bone marrow ward: “*we moved there only because of the negative pressure in the rooms…the downside was that the doors needed to be closed and we could not see and hear the patient, the same occurred with the aural alarms at the bedside; it was detrimental to patient safety*.“ As a countermeasure, both the patients and the screens displaying their vital signs were positioned so as they could be seen from the corridors through the small windows on the doors. Furthermore, two patients (sometimes male and female) used to be placed at the same room, compromising privacy despite the use of the mobile blind walls referred to in SP7. Nurse N1 pointed another possible drawback of these blind walls, namely the increased isolation of patients from their surroundings as a contributor to delirium.

Due to these drawbacks, the bone marrow ward was only used as a COVID ICU in the first pandemic peak, in April 2020. In the later pandemic stages this ward was also used as an ICU for non-COVID patients, releasing beds for COVID patients in the designed ICUs. In 2021, the surgical recovery unit was the only adapted COVID ICU. However, that unit had their own constraints, amplified by the sheer number of patients during the 2021 peak of infections. One of those constraints related to the proximity and impossibility of placing physical barriers between the beds. RDC 50/2002 establishes 1.0 m for bed separation in surgical recovery units, which is lower than the 2.0 m requirement for separation in ICU collective wards. Therefore, according to the interviewed doctor, patients could easily see and hear procedures like intubation being undertaken at the neighbouring patient, triggering feelings of anxiety: “*sometimes we did not know whether to assist the patient undergoing intubation or the patient crying on the neighbouring bed”*. Nurse N3 pointed out that “*the hospital management made the hard decision of keeping our doors open despite the degraded conditions…it was like war time, either this suboptimal condition or death.“*

### Slack in the electricity supply

Table 3 presents the identified instantiations of slack in the electricity supply infrastructure. Some of these applied to all hospital units (e.g., duplicated supply entry from the external grid to the hospital) and others existed in both the designed and adapted ICUs (e.g., spare batteries for certain equipment). According to the interviewees, existing slack coped effectively with the instabilities in the external electricity supply. This effectiveness is illustrated by the following report from Nurse N2: “*although our building was without electricity supply over 1.5 hour, the ICU was not affected owing to the no-break devices and extra batteries for life-supporting equipment…anyway, it was a stressful moment as we are not used to such combined failures* [external supply and hospital power generator had failed] *and the reliance on no breaks as the last barrier”.* Hospital´s records indicated that from March 2020 to November 2021 there were 97 failures in the external electricity supply (five a month, on average), either involving occurrences of undervoltage or complete power cuts – most had a very short duration, sometimes at the order of seconds. Nevertheless, given the patient safety implications, these records highlight the importance of multiple lines of defence.

**Table 3:** Slack in electricity supply

Code	Slack instantiation	N	Problems that created the need for slack	Designed / adapted ICU
SE1	Duplicated electricity supply entry from the external grid to the hospital	9	Instability in the external electricity supply	Both designed and adapted
SE2	No-break devices	9	Same as above	Designed. These devices were installed at the surgical recovery room in the later stages of the pandemic.
SE3	Availability of spare batteries to all life-supporting equipment	9	Same as above	Both designed and adapted
SE4	Power generators – two devices for the buildings where ICUs 1, 2, and 3 are located. One generator for the building where ICU 4 is located.	9	Same as above	Both designed and adapted
SE5	The number of power plugs was three-fold the regulatory requirement at ICUs 2, 3 and 4. In the adapted ICUs, the number of plugs at the early pandemic stages was equal to the minimum regulatory ICU requirement, even though additional plugs were installed later on.	10	Patients in critical condition needed more life-supporting equipment	Both designed and adapted
SE6	Aural warnings set to go off when using 70% of the maximum electricity load	4	Higher energy consumption and risk of short-circuits and fires – a principle of fire was reported in one of the designed ICUs	Designed
SE7	Availability of power plugs in corridors that link ICUs to other hospital units	1	Large number of patients transported from and to ICUs. These patients could need emergency care during the transportation.	Corridors that connect ICUs and other units

**N**: number of interviewees who reported the slack instantiation

**SE**: slack in electricity supply

The interviewees highlighted SE5, related to the large number of power plugs that proved to be crucial for the concurrent use of several life-supporting equipment. In fact, the need for extra power plugs was also a consequence of newer technologies and not only COVID, as remarked by nurse N3: “*old infusion pumps used to require only one plug, while some newer models require up to six plugs”*. However, the availability of power plugs was not uniform among the designed ICUs. Nurse technician NT1 stated that “*ICU 1, the oldest, does not have the same infrastructure…sometimes we do not have plugs for everything, we need to unplug something, do the exam, and the turn it on again*.“ Similar dissatisfaction was expressed by nurse technician NT3: “*there is a lack of plugs at ICU 1…some patients need 10 infusion pumps, which means 10 plugs*.“

The need for more electricity dependent equipment creates the need for slack in the sizing of the electrical circuits. Nurse technician NT2 was well aware of this situation: “*at ICUs 1, 2, and 3 (the three oldest), we cannot use certain combinations of machinery at the same time…there will be an overload and the circuits will fail*.“ As a result of the greater power demand, it was necessary to adjust the activation threshold for the aural warnings of electricity overload, as referred to in SE6. The two interviewed engineers revealed that the threshold was set closer to the load limit in order to prevent the constant activation of the alarms.

SE7, related to the installation of power plugs in corridors that link ICUs to other units, is worth highlighting due to its life-saving implications. The interviewed doctor commented that there were occasions of transporting patients under mechanical ventilation from the emergency department to the ICU and the battery of the ventilator went off during the trip. According to his report, “*the physical therapist who was following the patient immediately spot a plug and connected the ventilator…that was amazing, the person who thought of these plugs was illuminated*.“

Like the physical spaces, slack in the electricity supply for the adapted ICUs fell short of the designed ICUs. According to nurse N2, no-break devices did not exist at the bone marrow ward and, as a consequence, patients had their dialysis interrupted during a power outage. This nurse also described challenges in the surgical recovery room: “*there was no sufficient power supply to dialysis, leading the clinical team to think of how many patients under dialysis could be placed there*.“ Engineer E1 added that “*we had to make the clinicians aware of the maximum electrical load that could be used for each circuit, for each bed…awareness of the power capacity was crucial.“*

### Slack in the oxygen supply

Table 4 presents the identified instantiations of slack in the oxygen supply infrastructure. Some of these instantiations consisted of centralized hospital reserves (e.g., expanded storage capacity) and others played out the same way for both designed and adapted ICUs (e.g., distribution network with oversized pipes). Like the electricity infrastructure, oxygen supply was also dependent on an external supplier, which in this case had their own reserves that could serve as slack. The hospital is located at about 80 km from the supplier plant, making it possible to obtain deliveries 24/7, if necessary.

**Table 4:** Slack in oxygen supply

Code	Slack instantiation	N	Problems that created the need for slack	Designed ICU / Adapted ICU
SO1	Storage capacity of oxygen was expanded from 10,000 m^3^ to 30,000 m^3^	8	Very high demand of oxygen during the peaks of infections	Both designed and adapted
SO2	Valves for the regulation of pressure at the entrance of the ICUs allowed for increasing the oxygen flow rate	8	Same as above	Both designed and adapted
SO3	The main oxygen network was O-shaped. This contributed to the balance of pressures across the whole system and allowed for the replacement of one of the oxygen tanks without supply interruptions	8	Same as above	Both designed and adapted
SO4	All points of use could be used concurrently, having slack in relation to the 80% concurrent use established by RDC 50/2002	2	Same as above	Designed
SO5	There were four oxygen plugs per bed at ICUs 2, 3, and 4. This is twice the requirement of RDC 50/2002.	2	High number of oxygen plugs per bed was necessary for COVID patients	Designed
SO6	In all hospital units, the oxygen pipes had been designed with extra diameter and thickness, which facilitated the installation of extra plugs	8	Same as above	Both designed and adapted
SO7	Y-shaped components were installed at the oxygen plugs in order to create bifurcations and supply two patients from the same plug.	4	Same as above	Both designed and adapted

**N**: number of interviewees who reported the slack instantiation

**SO**: slack in oxygen supply

The demand for oxygen really challenged the hospital infrastructure only in the major pandemic peak of 2021, even though there was no lack of supply even during this period. According to the hospital records, the average oxygen consumption in 2019 was 1,070 m^3^/day. In March 2021, the daily demand was on average 5160 m^3^. Therefore, SO1, increasing storage capacity from 10,000 m^3^ to 30,000 m^3^, was crucial. On top of that, there were spare oxygen cylinders for the emergency resupply of the storage tanks, as mandated by regulation RDC 50/2002.

The remark as follows from Engineer E2 illustrates this point: “*during the peak, almost 100% of the ICU patients were intubated and were receiving oxygen at high flow rates…some of them were heavy and demanded the maximum possible flow rate…it was like working with all* [oxygen] *taps opened, with resupply twice a day”.* Thus, besides increasing the storage capacity, the oxygen pressure had to be adjusted in all ICUs, as indicated by SO2. Furthermore, it was easier to meet the demand in the designed ICUs as they had been planned for concurrent demand in all points of use – SO4. This means slack in relation to RDC 50/2002, which admits concurrent demand in 80% of the points of use.

Regulatory requirements were also met with slack in SO5, related to the number of supply points per bed. The documentary analysis revealed that, except for ICU 1 (it had two oxygen plugs per bed), the other designed ICUs had four oxygen plugs per bed, corresponding to 100% of slack in relation to the requirement of RDC 50/2002. Physical therapist P1 explained how the caregivers expanded the number of points of use at ICU 1: “*we installed a Y-shaped component at the oxygen plug, making a bifurcation and creating extra points.“* This adaptation (SO7) was also necessary, and made even more difficult, in the two adapted ICUs. For instance, the rooms of the bone marrow ward accommodated two patients each despite of being designed for a single patient. Thus, as described by Engineer E1, the Y-shaped components were accompanied by prolonging pipes and additional oxygen pressure and flow rate, in order to reach both beds.

### Slack in the air treatment

Table 5 presents the identified slack instantiations for the air treatment infrastructure. Similar to the other infrastructures, the amount of slack varied across the studied units, as acknowledged by nurse N3: “*we thought of many things related to ventilators, monitoring of vital signs, dialysis, but we overlooked the air quality…negative pressure was only possible in some units*”.

**Table 5:** Slack in air treatment

Code	Slack instantiation	N	Problems that created the need for slack	Designed ICU / Adapted ICU
SA1	Patient rooms at ICU 4 had exhaust fans and valves for pressure regulation that allowed changing air pressure from positive to negative	10	Air contaminated with the new coronavirus, demanding proper air filtering and renovation, besides negative pressure in the patient rooms	Designed ICUs
SA2	Air conditioning at ICUs 2, 3, and 4 had slack ranging from 100–300% in terms of air renovation capacity, in comparison to the requirement of RDC 50/2002	10	Same as above	Designed ICUs
SA3	Portable air filtering equipment were installed in the recovery room and marrow bone ward	9	Same as above	Adapted ICUs
SA4	Installation of doors and collective exhaust fans to isolate the recovery room from adjacent areas and create negative pressure	9	Same as above	Adapted ICUs

N: number of interviewees who reported the slack instantiation

SA: slack in the air treatment

The variations across the designed ICUs reflected the regulations, financial resources, and best practices at the time of design and construction. For example, the frequency of air renovation from the outside was eight times an hour in the regular patient rooms of ICU 4 (the newest), which means 300% slack in comparison to the RDC 50/2002 requirement of two renovations an hour. At this same ICU, the isolation rooms had air renovation 12 times an hour, implying 100% slack in comparison to the regulatory requirement. By contrast, at ICU 2, the regular patient rooms were limited to comply with the regulatory requirement (i.e., no slack) and the isolation rooms did not even match it. Regulations at the time of designing ICU 2 did not make any distinction between the frequency of air renovation for regular and isolation rooms. In the same vein, the weaknesses of ICU 1, the oldest, were evidenced by the interviewed doctor: “*the air at ICU 1 is the worst from all ICUs…COVID patients ideally should not have been placed there because there is no separate air flow between the patient rooms and the other areas such as the kitchen and nursing station; it is the same air*.“

As for air treatment in the adapted ICUs, the patient rooms in the bone marrow ward stood out as the transformation of the existing positive pressure into negative was technically simple and inexpensive. This was the main reason for using this ward in the first pandemic peak. The requirements for air quality in bone marrow wards, according to Brazilian standard NBR 7256/2021, are stricter than those for ICUs. However, it was not possible to fully reap the benefits of the superior air quality in that area, as recognized by all interviewees. This was due to the lack of visibility to the rooms from the outside, as described in Sect. 4.1. Therefore, caregivers needed to frequently enter the rooms for the monitoring of patients, being exposed to contaminated air. Physical therapist P1 described a resilient practice to counter the risk of infection: “*caregivers carried out multiple tasks once they entered the patient rooms…even if you were a doctor or a physical therapist you helped the nurse technician who was there, removing garbage, changing diapers…the intention was to reduce our exposition as well as reducing the frequency of donning and doffing PPE, saving it* “.

In the recovery rooms of the surgical unit, the other adapted ICU, air treatment was not as good as in the bone marrow ward. Slack instantiations SA3 and SA4 were attempts to minimize risks, by respectively involving the use of portable air filters and creating a large zone of negative pressure that covered all beds, isolating this area from the adjacent corridors through the installation of temporary walls. This large working zone shared by patients and caregivers came at a price to professionals as pointed out by nurse N4: “*there was no safe place to remove the face mask and drink water, our physical demand was much greater here in comparison to the designed ICUs”*.

## Discussion

The case study revealed 27 instantiations of slack in the infrastructures, encompassing both built-in slack (e.g., large patient rooms) and solutions devised on the spot by healthcare professionals (e.g., Y-shaped components added to the oxygen plugs). Several of these instantiations interacted with each other both *intra* and *inter* infrastructures. Interactions intra infrastructures seemed to be mostly linear, defined by [19] as those in expected and familiar sequences, quite visible, designed, and with proportionality between cause and effect.

These interactions intra infrastructures resemble a chain of resources that starts out of the ICUs and ends within their premises. We refer to the resources at the beginning of this chain as primary slack resources, also characterized by supporting all hospital buildings – e.g., duplicated electricity entry to the hospital complex, and extra storage of oxygen. Secondary slack resources are dedicated to a specific hospital building or set of buildings – e.g., generators for sets of buildings, and oversized electricity and oxygen distribution networks. Tertiary slack resources play out at the point of use at the ICUs such as the no-breaks to cope with power supply instabilities. This reasoning conveys that failures in the primary layer of defences can be absorbed by the second layer, then by the third layer, and so on. This notion of chain of slack resources is akin the principle of defences-in-depth [25], which promotes redundant barriers that separate a source of hazard from an undesired outcome. For ICUs, the application of this principle is recommended by authors who discuss the electricity and oxygen infrastructures. For example, [47] argue that a triple electricity entry should be considered for hospital complexes, while [11] recommend mobile emergency power generators, in addition to the static models observed in the case study. [33] set out similar proposals for oxygen supply. A chain of slack resources is logically related to two out of the four resilience potentials proposed by [5], namely: (*i*) the anticipating potential as these redundancies are designed well in advance of their actual deployment; and (*ii*) the responding potential plays out when there is an undesired variability (e.g., power outage) and the slack resource on standby is activated. These insights set the stage for the first proposition as follows:

### Proposition 1

to support resilient performance, interactions *intra* ICU infrastructures must be accounted for in the design of slack, starting from primary slack resources applicable to all hospital units down to the level of resources at the point of use.

The case study revealed that interactions inter infrastructures are also relevant. These are likely to be non-linear interactions, defined by [19] as those that occur in unplanned sequences, being either not visible or not immediately comprehensible, and with small causes that produce large consequences. The adapted bone marrow ward offered insight into this type of interaction as the benefits of slack in the air treatment installations were not fully exploited due to the inadequate layout of the unit. This example is consistent with previous studies of process improvement in health services, pointing out that individual improvements are not necessarily effective in face of unexpected interactions [48]. Against this background, the second proposition is set out below.

### Proposition 2

to support resilient performance, interactions *inter* ICU infrastructures must be accounted for in the design of slack, aiming at synergistic rather than conflicting relationships.

The weaknesses of the adapted units were also probably a source of stress for caregivers, both because their own health was at risk and because they worked under improvised conditions, adding to the normally stressful working conditions in ICUs [49]. In fact, more than in the designed ICUs, the accommodation of patients in the adapted ICUs was a matter of managing the trade-off between providing more beds and maintaining an acceptable infrastructure. Trade-off decisions are at the core of resilience management [50].

As such, the learning from the pandemic indicated that there should be designated units to be used as ICUs in case of need, counting on similar infrastructures. There were designated back-up areas for acute events such as fires but not for prolonged, chronic crises, such as the pandemic. Different types of disruptions imply in different patterns of system degradation and diverse slack resources [51]. Of course, this guideline is bounded by the financial slack of each hospital, which varies widely across countries and the public-private divide [52]. In this respect, there could be regulations establishing the minimum requirements for adapted ICUs. This would be an application of the idea that regulations, related to the macro level of healthcare systems, support resilient performance at the meso and micro levels [53]. Overall, the drawbacks of the adapted ICUs revealed the limits of the over-reliance on the resilient potential of responding, giving rise to the third proposition below.

### Proposition 3

in order to support resilient performance, hospitals should have designated areas as alternative ICUs and these should count on infrastructures as close as possible to the areas primarily designed as ICUs.

The complexities of the ICU infrastructures are also owed to the need for fulfilling the requirements of several stakeholders. The case study highlighted the complementary perspectives of clinical and engineering teams who worked in collaboration to detect the need for changes and devise solutions – e.g., at the early pandemic stages, the infrastructure team detected the rise of oxygen and electricity consumption and made the necessary changes to the possible extent. This positive use of diverse perspectives is crucial for coping with complexity and for supporting resilient performance [6]. It also demonstrates an application of the resilience potential of monitoring, as it implies several eyes paying attention to the infrastructures´ performance, triggering early warnings of insufficient slack. While collaborative work between healthcare professionals is widely discussed [54], much less attention has been devoted to the collaboration between caregivers and infrastructure engineers. These insights are encapsulated in the fourth proposition below.

### Proposition 4

in order to support resilient performance, the design of slack in the infrastructure of ICUs must consider both clinical and engineering perspectives.

Moreover, an overall implication of the case study concerns some of the requirements set out by the Brazilian regulations, which clearly did not suffice to cope with the pandemic. The same is likely to apply to regulations from other countries and guidelines from the World Health Organization (WHO). For example, [55] described how the provision of oxygen to severely-ill COVID patients in a Swedish district hospital differed widely from the WHO estimations, themselves not based on reliable empirical findings. Although the regulatory requirements do not prohibit the use of additional resources, in practice they bound decision-makers and provide legal protection. These findings suggest that the definition of slack as the portion of resources that exceeds the regulatory requirement is to some extent elusive. This point is reinforced by the diverse regulations in different countries, suggesting that what counts as slack is context-dependent [27]. At the same time, in face of evidence that a certain level of slack resources affects patient and staff outcomes, regulators should seriously consider changes in regulations. This would be consistent with the idea that certain aspects of the built environment are equivalent to medical interventions, requiring ethical scrutiny and empirical study [56]. Proposition 5 is a consequence of these reflections.

### Proposition 5

some requirements of Brazilian regulations for slack in the infrastructure of ICUs are too low for supporting resilient performance and must be revised.

Despite the aforementioned propositions and associated insights, some limitations of this research must be highlighted. First, findings derived from the case study of a leading private hospital, which was subject to lower financial constraints in comparison to other healthcare organizations. Lower resource organizations are likely to find it harder to manage the trade-off between safety and efficiency in favour of safety, being pressed to reduce slack. In this same vein, a second limitation relates to the lack of a cost-benefit analysis of investing in slack. Stricter regulatory requirements can make this financial analysis less relevant as slack becomes mandatory. Third, we did not investigate the association between patients´ outcomes and whether the ICUs were designed or adapted. Due to the lower slack resources in the adapted ICUs, the hypothesis is that patients hospitalized in those areas had worse outcomes, posing ethical dilemmas for hospital managers. In fact, patient outcomes are hypothesized to have varied even across the designed ICUs as the older ones were built under less strict regulations. Fourth, slack in terms of staff and human resources in general was out of the study´s scope, although they are crucial for the effective use of the slack in the technical infrastructures.

## Conclusions

The research question that guided this study referred to how the design of slack in the infrastructure of ICUs can support their resilient performance in the post-pandemic era. This question was investigated in the context of infrastructures related to physical space, electricity supply, oxygen supply, and air treatment. This investigation gave rise to propositions addressing: interactions intra and inter infrastructures; the need for adapted ICUs that match as closely as possible the designed ICUs; the consideration of both clinical and engineering perspectives in design; and the need for the revision of some requirements of the Brazilian regulations. The propositions answer the research question, offering novel insights into the design of ICUs, using the lens of resilience engineering. These contributions are relevant to the designers of the infrastructures, to the designers of clinical activities, and to top management who is the ultimate responsible for decision-making on whether or not to invest in slack. The pandemic dramatically demonstrated the value of investing in slack resources, creating momentum for this discussion in health services. This investment is not only important to cope with pandemics but also with other threats such as ageing populations and natural disasters. Several instances of slack (e.g., duplicated electricity supply) tend to be useful in a wide variety of circumstances.

Given the design implications of our findings, they contribute mostly to the resilience potential of anticipating, which is often the least developed potential in health services. The potential of responding also benefits once the slack resources are activated in practice. The monitoring potential manifests on the spot as the crisis unfolds, as both the engineering and clinical teams detect, as early as possible, the need for adjustments in the slack. Further, our study indicated that learning from the pandemic sets a basis for anticipation and better preparedness for future similar events.

Opportunities for further studies resulted from this research such as: (*i*) to carry out a similar investigation in hospitals with different characteristics, providing a stronger basis for the revision of regulatory requirements; (*ii*) to conduct a similar study in hospital areas such as emergency departments and surgical units, and also considering other infrastructures such as water supply; (*iii*) to investigate the association between slack and patient outcomes; (*iv*) to investigate the association between slack and professionals´ safety and well-being; (*v*) to assess the cost-benefit of providing slack, considering both financial and non-financial measures; (*vi*) to analyse the interactions between slack in the infrastructures and slack related to human resources; and (*vii*) to include slack as a performance dimension in frameworks for the evaluation of service quality. These future studies are relevant to both the pandemic and the non-pandemic contexts.

## Electronic supplementary material

Below is the link to the electronic supplementary material.

Supplementary Material 1

## Data Availability

Documents retrieved from online sources are publicly available. Documents exempted from public disclosure are not available. Data retrieved from the interviews is available from the corresponding author upon request and with permission from the participant(s).

## Footnotes

1 Hereafter referred to simply as the pandemic.

2 Provision of slack resources is relevant to address clause 6.1 of ISO 9001/2015. This clause requires organizations to implement a process to identify, determine, and evaluate risks and opportunities related to quality management systems performance [40].

3 As an illustration of the leadership role of the selected nurses, N1 was a major source of information to the local media on how the hospital ICUs coped with pandemic.
