# Supplementary material for: Slack in the infrastructure of intensive care units: resilience management in the post-pandemic era
Source: BMC Health Serv Res. 2023 Jun 6;23:579. doi: 10.1186/s12913-023-09495-4 (PMC10241554; doi:10.1186/s12913-023-09495-4)
Supplement: Supplementary file 1 — Supplementary Material 1 [file 12913_2023_9495_MOESM1_ESM.docx]

**Supplementary file 1**: question guide for the semi-structured interviews with healthcare professionals

**Respondent profile**

Gender, age, job, professional experience in healthcare in general, ICU experience, brief description of the respondent´s daily activities.

**Slack in the ICU infrastructures**

1) Please describe how the physical space (e.g., layout, size, furniture) of the ICUs affected your activities during the pandemic. Could you describe specific instances when the physical space either hindered or facilitated patient care and daily activities?

2) Please describe how the electricity supply at the ICUs affected your activities during the pandemic. Could you describe specific instances when the electricity supply either hindered or facilitated patient care and daily activities?

3) Please describe how the oxygen supply at the ICUs affected your activities during the pandemic. Could you describe specific instances when the oxygen supply either hindered or facilitated patient care and daily activities?

4) Please describe how the air treatment at the ICUs affected your activities during the pandemic. Could you describe specific instances when the air treatment either hindered or facilitated patient care and daily activities?
